# Adolescent Life Satisfaction: Association with Psychological, School-Related, Religious and Socially Supportive Factors

**DOI:** 10.3390/children10071176

**Published:** 2023-07-07

**Authors:** Bettina F. Piko

**Affiliations:** Department of Behavioral Sciences, University of Szeged, 6720 Szeged, Hungary; fuzne.piko.bettina@med.u-szeged.hu; Tel.: +36-62420530

**Keywords:** life satisfaction, subjective well-being, depression, future orientation, social support, family, SES self-assessment, religiosity

## Abstract

Adolescent life satisfaction is crucial to later adult health and well-being; therefore, searching for its correlates should receive priority in research. The aim of this study was to explore the role of psychological (depression, future orientation), school-related (school achievement, satisfaction with school), religious (going to church, importance of religion), socially supportive (family and friend support), other familial, and sociodemographic (age, sex, self-assessed socioeconomic status) factors in adolescent life satisfaction. This cross-sectional survey (entitled Szeged Youth Study 2022) involved a sample of middle and high school students (N = 2239, aged 11–18 years, 51.8% females) from public schools in Szeged, Hungary. Besides descriptive statistics, correlation and multiple regression analysis were applied to the data analyses. Boys scored higher on the life satisfaction scale (Satisfaction with Life Scale, SWLS), while the level of depression (Children’s Depression Inventory, CDI) was higher among girls. In the final regression model, family support was the strongest predictor of life satisfaction (*β* = 0.44, *p* < 0.001), followed by depression (as a negative contributor), socioeconomic (SES) self-assessment, future orientation, satisfaction with school, going to church, and friend support. School prevention programs should be focused not only on preventing mental health difficulties but also promoting adolescent well-being.

## 1. Introduction

Recently, considerable attention has been paid to subjective well-being; this includes life satisfaction among adolescents, since a number of biological and psychosocial changes happen in this life period that may have an impact on it [1]. This is particularly relevant since adolescent well-being is crucial in later adult mental health [2]. During adolescence, mental health and well-being tend to deteriorate [3]. While more studies concentrate on substance use, health risk behaviors, and mental health problems, searching for protective factors in order to promote positive development and strengthen well-being would be extraordinarily important [4]. 

Well-being has a great relevance in maintaining positive mental state and preventing psychological disturbances and psychiatric diseases (e.g., depression or anxiety). Although there is no consensus on a definition of well-being, there is a broad and detailed description: “Well-being has been defined as the combination of feeling good and functioning well; the experience of positive emotions such as happiness and contentment as well as the development of one’s potential, having some control over one’s life, having a sense of purpose, and experiencing positive relationships” [5]. While feeling good is closer to the hedonistic viewpoint, functioning well represents the eudaimonic aspect of well-being. Subjective well-being can also be viewed as synonymous with positive mental health [5] based on the definition of the WHO: “a state of well-being in which the individual realizes his or her own abilities, can cope with the normal stresses of life, can work productively and fruitfully, and is able to make a contribution to his or her community” [6]. There is an argument, however, that well-being and mental health are not necessarily related and only a moderate overlap exists between the two domains [7]. This means that some individuals with mental disorders may experience good well-being, while those without mental health problems may not be happy with their lives. As a consequence, preventing mental disorders is not unequivocally the same as improving one’s well-being. Therefore, this study is focused on subjective well-being but also includes negative mental health as a possible correlate.

Some researchers focus on specific components of well-being, such as happiness, positive functioning, or positive emotions, while others suggest a cohesive, multidimensional measure of well-being. A single subjective evaluation is also widely used in studies [8]. Among the indicators of subjective well-being, life satisfaction is a frequent measure of subjective well-being. As a global assessment of a person’s quality of life, it reflects a positive orientation towards one’s life in general [9]. Previous studies found that levels of life satisfaction declined during adolescence [10], and females reported lower levels; however, this difference does not persist in adulthood [11]. 

Unsurprisingly, since life satisfaction is a positive construct, the negative emotions and psychological states, such as hopelessness or depressed mood, are often negatively correlated with it [12,13]. Depression is a common mental health problem in adolescence, with a surplus among girls [14], and even subclinical depressive symptoms may have a great impact on well-being [15]. 

It seems that satisfaction (or dissatisfaction) with current life circumstances is not the only characteristic of well-being and mental health, but also how the individual looks into the future. Depressed youth tend to have a feeling of worthlessness and hopelessness, without positive prospects for the future. On the other hand, life satisfaction, as a positive construct, is greatly related not only to satisfaction with current life circumstances but also to a positive view about the future and the ability to plan for the future [16]. Older adolescents, in particular, are better able to consider future plans and the consequences of present decisions [17]. While depressive symptoms (e.g., sadness and hopelessness) deteriorate one’s ability to plan for a positive future, life satisfaction is associated with positive future orientation and future time perspectives [18]. In a word, life satisfaction, depression, and future orientation seem to be relevant aspects of adolescent development. As a result, this study also includes adolescent future orientation as a possible contributor to life satisfaction. 

Besides these cognitive and affective aspects of well-being, at this age, social network also has a decisive role in adolescent development, and social relationships can have a great influence on youth life satisfaction. During adolescence, there is a significant restructuring of the social network: adolescents’ striving for autonomy leads to an increase in peer-based activities. While spending leisure time with peers is often found to elevate the risk of substance use [19], socializing with peers seems to be an important process in adolescent social development [20,21]. However, despite the increased orientation towards peers, parents remain a relevant source of protection for youth. Parental supervision often seems to evoke negative attitudes in adolescents, but it functions as a protective factor against adolescent substance use and problem behaviors [22,23]. Social support from the family and shared activities (e.g., common meals, cultural events, or excursions) can strengthen family cohesion [24,25]. Family serves as a field of primary socialization for child development, providing values, norms, behavioral guidelines and mindset, lifestyle choices, and well-being in the long term [26,27]. All in all, the family still plays a positive role in adolescents’ life satisfaction [25,26,28,29]. 

The family environment also includes family affluence and other socioeconomic circumstances and contributes to life satisfaction through, for example, self-esteem and optimism [28,30]. While objective socioeconomic status (SES) measures usually assess various indices of material wealth, such as income or occupation, subjective SES is primarily based on a comparative evaluation of one’s own financial situation and possibilities; unsurprisingly, this latter variable is a better indicator for predicting subjective well-being [31]. While the accuracy of children’s proxy reports of parents’ objective SES is questionable and often goes together with a high rate of non-responders, the self-evaluated (subjective) SES measure seems valuable and informative in research: it has been found to be a predictor of not only psychological states but also psychosomatic and physiological outcomes [32,33]. 

Besides family and peers, the school has an important role in socialization, providing knowledge of the world and teaching behavioral guidelines. This is particularly relevant in terms of the children’s well-being since they spend a lot of time in school. Unsurprisingly, among the contributors to adolescent life satisfaction, school-related variables also occupy a top position. In particular, school climate, school attachment, and how they feel about school in general can act as protective factors against adolescent problem behavior and enhance quality of life [34]. Although school achievement is often viewed as a marker of school success, school satisfaction and enjoyment of the learning environment seem to be more effective elements of positive schooling and child development [35].

Finally, religion was found to play a positive role in children’s socialization and their mental health [36]. Despite a decrease in the importance of religiosity, frequency of service attendance, and praying frequency among Christian youth [37], religiosity—often originated in the family—has been found to play a beneficial role in child development and adjustment [38]. For example, in a sample of Italian adolescents, religiosity was found to be related to life satisfaction, both directly and indirectly, mediated by positivity [39]. Rates of substance use also tend to be lower among religious youth [40]. Religiosity can act as a protective factor for child development in a number of ways, for example, providing a comprehensive value system, religious coping, or going to a religious community; all these aspects may enhance adolescents’ subjective well-being.

In this study, the aim was to explore adolescents’ life satisfaction, including a set of variables known to contribute to their well-being in one model. Beyond sociodemographic factors (age, sex, SES self-assessment), we investigated the role of socially supportive factors (friend and family support, parental supervision, shared meal with the family), religious and school-related factors, as well as positive (future orientation) and negative (depression) psychological variables. We detected both bivariate relationships between life satisfaction and these variables and applied multivariate analysis to find the most relevant predictors. Based on previous theoretical considerations and research results, we anticipated that future orientation, social support from family and friends, other family-related variables (parental supervision, shared meals), and religious and school-related factors would be positive correlates of adolescent life satisfaction, while depression would be a negative correlate. In terms of sociodemographic factors, we hypothesized that age and being a female would be negative predictors, while SES self-assessment would be a positive predictor. 

## 2. Materials and Methods

### 2.1. Participants and Procedure

Participants of this survey were middle and high school students (from 7th to 12th grades, age range between 12 and 18 years) in Szeged, Hungary, as part of the Szeged Youth Study 2022 organized by the Local Collaborative Forum for Drug Issues (Kábítószerügyi Egyeztető Fórum, KEF in Hungarian). The aim of the Szeged Youth Studies is to obtain actual information on health-related issues of the student population from this age group. Based on these data, several health education and training programs will be developed in which the enhancement of health and well-being will receive priority. The study population was students of this age group from public schools in Szeged, including 9520 students altogether (from 21 primary and 21 high schools), of which one quarter (25%) were randomly selected and asked to participate. Thus, the total number of students sampled was 2380, of which 2239 returned (aged between 12 and 18 years, mean = 14.61 years, SD = 1.71; 51.8% females), yielding a response rate of 94%. This research and all study procedures were approved by the Institutional Review Board (IRB) of the Doctoral School of Education, University of Szeged, Hungary. The survey was carried out in the autumn semester of the school year 2022–2023. Data were collected in the computer labs during different classes for the students through an online survey using Google Drive forms with the help of local teachers. Participation in the study was voluntary and anonymous. Prior parental informed consent was obtained in all cases: a written form was sent to parents and asked for their children’s participation. The questionnaire took approximately 25–30 minutes to complete.

### 2.2. Measurements

The questionnaire contained measurements on psychological variables, socially supportive factors, religiosity, and school-related variables, in addition to sociodemographic factors.

#### 2.2.1. Sociodemographic Factors

With regard to sociodemographic factors, the following data were collected: age, sex, and SES self-assessment. SES self-assessment is a subjective evaluation of the family’s financial situation compared to an estimated average. The evaluation was coded on a 7-point rating scale from ‘among the worst’ (1) to ‘highly among the best’ (7). This measure was derived from ESPAD (European School Survey Project on Alcohol and Other Drugs) for the purpose of comparison [41]. 

#### 2.2.2. Psychological Scales

Life satisfaction was measured by the Hungarian validated version [42] of the Satisfaction with Life Scale (SWLS) [9]. This measurement assessed a global evaluation of life satisfaction as an indicator of subjective well-being. Participants indicated how strongly they agreed with each of the five items (e.g., “The conditions of my life are excellent.”), with responses ranging from 1 (strongly disagree) to 7 (strongly agree). Higher scores reflect higher levels of life satisfaction. Cronbach’s alpha coefficient of reliability was 0.89 with the current sample.

Depression was measured by a validated, shortened version [43] of the original 27-item Children’s Depression Inventory (CDI) [44]. Each item of the original and shortened versions assesses a single symptom, such as sadness or hopelessness, and they are coded from 0 to 2. We weighted the shortened CDI by a factor of 3.375 (number of original CDI items 27/shortened version items 8 = 3.375) for the purpose of comparison. Based on the present data, the scale was reliable, with a Cronbach’s alpha of 0.84 (mean = 10.64; SD = 10.58).

Future orientation was measured by an adapted and shortened Hungarian version [45] of the Consideration of Future Consequences Scale [46]. The scale consisted of six statements (e.g., “I am willing to sacrifice my immediate happiness or well-being in order to achieve future outcomes”). The respondents indicated the extent to which each statement was characteristic of their attitudes from 1 (extremely uncharacteristic) to 5 (extremely characteristic). The final results were coded from 6 to 30; a high CFC score indicates a high degree of importance placed on the future consequences of a behavior. Cronbach’s alpha coefficient with this sample was 0.70. Despite this scale not having been validated yet on Hungarian adolescents, it is widely used, and the reliability value was similar to the original samples [45].

#### 2.2.3. Socially Supportive Factors

Social support from family and friends was measured by using the Multidimensional Scale of Perceived Social Support (MSPSS) [47], the Hungarian validated version [48]. Both subscales contained four items, for example, “I can talk about my problems with my family” (family support) or “I can talk about my problems with my friends” (friend support). Adolescents were asked to indicate how strongly they agreed with each statement on a five-point Likert-type scale. Higher scores suggested more social support. These subscales were reliable with the following Cronbach alphas: family support, α = 0.93; friend support, α = 0.95.

#### 2.2.4. Familial Factors

Parental supervision was measured by a summary score of two items: whether the parents set a curfew and whether they know where their children are when going out with friends [49]. Responses vary from 1 (never) to 4 (always). In addition, we also measured frequencies of having an evening meal together as a family with similar response categories. These items were adapted from previous Szeged Youth Studies [49]. 

#### 2.2.5. School-Related Factors

Two school-related variables were included: school achievement and how the adolescents feel about their school. Both questions, derived from previous Szeged Youth Studies [49], were measured on a seven-point rating scale. For school achievement, we asked the students: “What grades do you mostly get in school?” Responses vary from 1 (mostly Ds and Fs, which is equivalent with 1 and 2 in the Hungarian school system) to 7 (mostly As, that is, 5). For the question “How happy are you with school right now?” the responses varied from 1 (not at all) to 7 (very much). 

#### 2.2.6. Religious Factors

First, as a measure of religious activity, frequency of going to church was measured. This variable was dichotomized (1 = rarely or only on festive occasions, and 2 = regularly). The second questions dealt with how important religion was for the respondents, with responses ranging from 1 (not at all) to 7 (very much) [41]. 

### 2.3. Data Analysis

The program SPSS for MS WINDOWS Release 25.0 was used in the calculations, with a maximum significance level set at 0.05. First, descriptive statistics were calculated for the study variables by sex, where Student’s *t*-test and chi-square test were applied to determine significance. In parallel with this, correlation coefficients were calculated to test bivariate relationships. Finally, multiple regression analysis (stepwise method) was used to detect the order of the most relevant predictors of adolescent life satisfaction. 

## 3. Results

### 3.1. Descriptive Statistics 

Table 1 presents descriptive statistics for study variables by sex. Boys scored significantly higher on the life satisfaction scale (t(2237) = 6.18, *p* < 0.001) and reported higher levels of family support (t(2237) = 5.48, *p* < 0.001). Girls reported more depressive symptoms (t(2237) = −16.32, *p* < 0.001), received more parental supervision (t(2237) = −5.39, *p* < 0.001), and received more support from their friends (t(2237) = −7.81, *p* < 0.001). More boys 51.5%) than girls (46.1%) shared evening meals with their families (χ^2^ (1) = 6.54, *p* < 0.05). There was no sex difference in frequencies of regularly going to church (16.3% of boys vs. 14.8% of girls; *p* > 0.05). Regarding descriptive statistics of sociodemographic factors, the mean age of the sample was 14.6 years (SD: 1.71). The distribution of SES rating was similar to the last Hungarian ESPAD survey (average or above to some degree: 67.6% in our sample vs. 70.1% in the national representative sample; below the average: 4.0% vs. 5.2%; better than the average: 28.3% vs. 24.7%).

### 3.2. Correlation Analysis 

Table 2 shows the correlation matrix, with results for boys above the diagonal and girls’ results below. Among boys, life satisfaction was positively correlated with future orientation (*r*(1080) = 0.42, *p <* 0.01), parental supervision (*r*(1080) = 0.22, *p <* 0.01), having dinner together with the family (*r*(1080) = 0.25, *p <* 0.01), social support from the family (*r*(1080) = 0.65, *p <* 0.01), friend support (*r*(1080) = 0.45, *p <* 0.01), school achievement (*r*(1080) = 0.17, *p <* 0.01), satisfaction with school (*r*(1080) = 0.32, *p <* 0.01), importance of religion (*r*(1080) = 0.07, *p <* 0.05), going to church (*r*(1080) = 0.10, *p <* 0.01), and SES self-assessment (*r*(1080) = 0.22, *p <* 0.01). Its relationship with depression was negative (*r*(1080) = −0.48, *p <* 0.01). Similar to boys, girls’ life satisfaction shows the strongest relationship with family support (*r*(1159) = 0.70, *p <* 0.01), and the association with depression was also considerably strong (*r*(1159) = −0.62, *p <* 0.01). We should highlight the negative relationship between depression and family support in both sexes: *r*(1080) = −0.41, *p <* 0.01 for males and *r*(1159) = −0.54, *p <* 0.01 for females. With growing age, not only did life satisfaction tend to decrease but also parental supervision, having shared meals with the family, social support from the family, and religiosity. SES self-assessment also went together with other sources of protection, such as social support, having a shared meal or school achievement, and satisfaction with school. 

### 3.3. Multiple Regression Analysis (Stepwise Method)

Finally, we applied multiple regression analysis (stepwise method) to detect the order of the most relevant predictors of adolescent life satisfaction (Table 3). We preferred this method to involve a selection of independent variables to be used in the final model.

Among the independent variables, social support from the family proved the most relevant predictor (*β* = 0.44; *p* < 0.001). Next, depression as a negative predictor occupies the second place (*β* = −0.24; *p* < 0.001). Third, SES self-assessment was a positive contributor to life satisfaction (*β* = 0.11; *p* < 0.001). Other significant but less relevant predictors were the following: future orientation (*β* = 0.09; *p* < 0.001), satisfaction with school (*β* = 0.08; *p* < 0.001), going regularly to church (*β* = 0.05; *p* < 0.05), and friend support (*β* = 0.05; *p* < 0.05). These variables together explained 55% of variance. The reliability of the model was further examined with VIF (variance inflation factor) indices and tolerance values. Collinearity statistics justified independence among the variables: the VIF values were all within the acceptable range (below 2) and values of tolerance >0.60 (see Table 3). Parental supervision, shared dinner, importance of religion and school achievement, age, and sex were excluded from the final regression model (*p* > 0.05). 

## 4. Discussion

Adolescence is a life period of tremendous biological and psychosocial changes, and there is a declining tendency in mental health and well-being among youth [3]. Adolescent life satisfaction is crucial to later adult health and well-being [2]; therefore, searching for its correlates should receive priority in research. Potential protective factors may help neutralize these negative processes in mental health. This study aimed to explore the role of psychological, school-related, religious, and socially supportive factors in adolescent life satisfaction in order to find the most relevant contributors. Social support from the family seemed the most important predictor, followed by lower levels of depression, higher score on the SES self-assessment scale, positive future orientation, greater satisfaction with school, going to church, and friend support. While other variables were also significant in bivariate relationships with life satisfaction, they became nonsignificant in multivariate analysis. 

Our findings suggest that social support from the family is the strongest protective factor for adolescent well-being. Although adolescence is the life period where children’s autonomy increases and peers become a more decisive part of the adolescents’ social network, the family remains an important source of protection regarding adolescent well-being and health behaviors [22,23,25,26,28,29]. The need for parental guidelines is still inevitable, and they contribute to adolescent life satisfaction if they are functioning in a proper way [23,25,26,27]. Social support from the family can serve as social capital for the members, providing emotional or instrumental support, guidance, acceptance, and understanding [25,47]. Even providing financial help is a fundamental part of social support, and the family may be the dominant resource for this, particularly in this age group. Unsurprisingly, SES self-assessment was significantly and positively correlated with social support from the family. Furthermore, family support was negatively related to adolescent depression, suggesting that it may serve as protection against mental health problems. Since cause-and-effect relationships cannot be justified in a cross-sectional study, it may also mean that a lack of family support can contribute to adolescent depression. Although parental supervision and shared meals with the family became nonsignificant in the multivariate analysis, they were significantly correlated with family support, indicating the importance of these factors in family cohesion [22,23,24,25].

Depression was the second strongest predictor of life satisfaction, while future orientation was also a significant but less strong predictor. The close connection between life satisfaction and depression is in concordance with previous findings [12,13,15]. Depressive symptoms deteriorate positive attitudes towards life [12,50], as well as toward the future [16]. Although a previous study has suggested that mental health is not necessarily associated with well-being [7], our result supports the opposite. When individuals experience negative mood states, they become less satisfied with their lives and tend to see positive circumstances or relationships negatively (that is, negativity bias) [50].

In contrast with depression, future orientation was a positive contributor. A positive attitude toward the future in childhood is a drive for planning and striving, which is very beneficial for child development and well-being [16,17,18]. Future orientation showed a positive association with life satisfaction and a negative association with depression in our study. In addition, while future orientation was in a positive relationship, depression was in a negative relationship with all the social support variables and a low level of satisfaction with school. These associations support the protective role of future orientation. 

Among sociodemographic variables, age was slightly and negatively correlated with life satisfaction, and its level was higher among boys; however, these variables became nonsignificant in the final multivariate model. On the other hand, SES self-assessment was the third strongest predictor of adolescent life satisfaction. The positive role of subjective SES ranking in adolescents’ well-being and health has been well documented [32,33]. We should not forget that living in a consumer culture makes material values desirable, and thus having material possessions can contribute to satisfaction with life. The social and socioeconomic situation of the adolescent’s family is associated with well-being [28,30], not only in terms of the objective financial situation but also its subjective evaluation. 

As one of the school-related factors, satisfaction with school was a significant contributor to adolescent life satisfaction. In addition, it seemed more important than school achievement. The school environment can contribute to positive child development in many ways (e.g., building fruitful social network or providing creative activities, meeting with peers, etc.) [34,35]. All of these may also compensate for problems around school achievement and strengthen life satisfaction.

Religiosity has been always viewed as a protective factor in a classical sense [36,38]. Even in secularized societies, religious variables are often found to play a protective role in adolescent well-being [38,39] and substance use [40]. In our sample, only 16.3% of males and 14.8% of females reported going to church regularly (and not only on festive occasions), but it contributed to life satisfaction among these adolescents. This finding is similar to a study among Italian adolescents [39]. However, the low level of going to church also supports previous research results on the declining importance of religion in Europe [37]. 

Finally, social support from friends was also among the significant predictors. In bivariate analysis, it was positively correlated with family support; however, its role in adolescent life satisfaction was much lesser. Although peer-based relationships are becoming more and more important for children during adolescence, and their role in social development is crucial [21], the pivotal protection still stems from the family. 

Among the strengths of this study, we should mention the large sample size with a relatively wide age range and the representativeness of the sample, as well as the application of mostly validated and previously adapted scales and measurements in our survey. The most important significance of our analysis is that these results add further information on adolescent life satisfaction, including psychological, familial, school-related, and religious factors as well as socially supportive relationships in one model. However, some limitations also need to be considered. First, due to the cross-sectional design, our results cannot provide any cause-and-effect relationships. Second, our data is based on only the self-reported prevalence of depression and other attitudes and social activities, which may be biased; however, online data collection ensures confidentiality and anonymity for the students. There are debates about the use of online sampling, but young people’s preference for online platforms helps to obtain data that are valid and reliable [51]. Finally, our sample excluded adolescents between 16 and 18 years old who already left school and those went to private/parochial/nonstate schools. Our list of variables may also lack some other relevant factors (e.g., health-related variables), and these should be included in further research.

Finally, the following future research directions are recommended. First, certain variables need more exploration: for example, more family-related factors should be included, such as parenting style and family cohesion. Although religion may act as protection, we should clarify adolescent religion and spirituality in greater details. Besides depression, other mental health difficulties could also be included (e.g., anxiety or attention disorders). There is also a need to better understand the relationship between adolescent well-being and youth’s digital device use; however, this would be another large-scale study. Second, a longitudinal study design should be applied to test changes of these variables over time. 

## 5. Conclusions

Overall, the results of this study highlight some important correlates of adolescent life satisfaction as a valuable indicator of subjective well-being. Above all, social support from the family seems to be the most relevant resource of life satisfaction. Although friend support seems to play a lesser role, it can also contribute to adolescents’ well-being. While a higher level of depression may deteriorate life satisfaction, positive future orientation can enhance life satisfaction. Higher rank on the SES self-assessment scale is correlated with greater satisfaction with one’s life due to better life circumstances as perceived by the adolescent. Going to church regularly can serve as protection for those who reported religion being important in their lives. School environment can enhance adolescents’ well-being if they are satisfied with school. 

Based on our results, several important points need to be taken into consideration for interventions. Adolescent life satisfaction is crucial to healthy development, which is a great challenge in modern society with many risks and threats to mental health. This is particularly true in a life period with rapid biological and psychosocial changes. Mental health promotion in schools should include training for social and communication skills, which may be useful in the prevention of mental disorders and enhancement of well-being. In addition, this training would also be fruitful for building social networks and strengthening social support. Family involvement is essential in these programs to build on social support from family members. This training should be focused on learning techniques to achieve a positive mindset. A great advantage of this viewpoint is that it involves every student, not only those with mental health difficulties; as a result, we can prevent unnecessary labeling [52]. Students should learn to recognize their mood states and learn techniques for how to cope with them. A comprehensive school health framework for student mental health and well-being can be effective only in collaboration with families and communities [53]. Child psychiatrists and school psychologists can provide valuable guidance for a school mental health implementation team. Modern digital devices may also be applied during these interventions since digital interventions have the potential to promote adolescent mental health but only with strong professional guidance [54]. While adolescent life satisfaction has an impact on later adult health and well-being [2], this is also a life period where the children are responsive to preventive messages. 

## Figures and Tables

**Table 1:** Descriptive statistics for study variables by sex (N = 2239).

Variable (Scores)	Range	Skewness (SE)	Kurtosis (SE)	M ± SD Males	M ± SD Females
1. Satisfaction with life *	5–35	−0.59 (0.05)	−0.48 (0.10)	25.13 ± 7.52	23.15 ± 7.60
2. Future orientation	6–30	−0.53 (0.05)	1.18 (0.10)	20.31 ± 4.63	20.23 ± 4.38
3. Depression (CDI) *	0–54	1.22 (0.05)	1.41 (0.10)	7.07 ± 8.43	13.97 ± 11.27
4. Parental supervision *	2–10	−0.57 (0.07)	−0.040 (0.15)	7.15 ± 2.29	7.86 ± 2.13
5. Family support *	4–28	−1.06 (0.05)	0.09 (0.10)	22.59 ± 6.30	21.03 ± 7.10
6. Friend support *	4–28	−1.28 (0.05)	0.82 (0.10)	21.48 ± 6.59	23.56 ± 5.98
7. School achievement	1–7	−0.72 (0.05)	0.16 (0.11)	5.11 ± 1.23	5.23 ± 1.22
8. Satisfaction with school	1–7	−0.36 (0.05)	−0.21 (0.10)	4.34 ± 1.5	4.23 ± 1.39
9. Importance of religion	1–7	0.85 (0.05)	−0.45 (0.10)	2.69 ± 1.86	2.67 ± 1.84
10. Age	12–18	0.50 (0.05)	−0.68 (0.10)	14.67 ± 1.74	14.54 ± 1.68
11. SES self-assessment	1–7	−0.01 (0.05)	0.25 (0.10)	4.98 ± 0.97	4.84 ± 1.04

Note. * *p* < 0.001. Student’s *t*-test. SD, Standard Deviation; SE, Standard Error; CDI, Child Depression Inventory; SES, Socioeconomic Status.

**Table 2:** Zero-order correlation matrix for study variables by sex (N = 2239).

Variable (Scores)	1	2	3	4	5	6	7	8	9	10	11	12	13
1. Satisfaction with life	-	0.42 **	−0.48 **	0.22 **	0.25 **	0.65 **	0.44 **	0.17 **	0.32 **	0.07 *	0.10 **	−0.15 **	0.23 **
2. Future orientation	0.34 **	-	−0.19 **	0.08	0.09 **	0.38 **	0.39 **	0.06 *	0.16 **	0.07 *	0.09 *	0.01	0.07 *
3. Depression (CDI)	−0.62 **	−0.23 **	-	−0.24 **	−0.19 **	−0.41 **	−0.24 **	−0.12 **	−0.36 **	−0.04	−0.06 *	0.01	−0.10 **
4. Parental super- vision	0.30 **	0.17 **	−0.27 **	-	0.22 **	0.29 **	0.08	0.10 *	0.16 **	0.08	0.06	−0.32 **	0.04
5. Shared dinner (most of the time)	0.26 **	0.10 **	−0.21 **	0.19 **	-	0.24 **	0.10 **	0.07 *	0.14 **	0.15 **	0.15 **	−0.17 **	0.16 **
6. Family support	0.70 **	0.32 **	−0.54 **	0.37 **	0.28 **	-	0.48 **	0.13 **	0.29 **	0.05	0.09 **	−0.18 **	0.13 **
7. Friend support	0.38 **	0.27 **	−0.39 **	0.22 **	0.12 **	0.35 **	-	0.04	0.19 **	0.05	0.06 *	0.04	0.07 *
8. School achievement	0.25 **	0.18 **	−0.22 **	0.24 **	0.10 **	0.24 **	0.13 **	-	0.25 **	0.06	0.09 **	−0.11 **	0.09 *
9. Satisfaction with school	0.38 **	0.18 **	−0.46 **	0.19 **	0.17 **	0.27 **	0.26 **	0.21 **	-	0.06 *	0.08 **	−0.05	0.10 **
10. Importance of religion	0.12 **	0.07 **	−0.03	0.12 **	0.09 **	0.10 *	−0.03	0.04	0.09 **	-	0.59 **	−0.13 **	0.04
11. Going to church	0.04	0.01	0.03	0.08	0.07 *	0.01	−0.10 **	0.05	0.06	0.58 **	-	−0.11 **	0.01
12. Age	−0.08 **	−0.01	0.02	−0.30 **	−0.08 *	−0.08 *	−0.06 *	−0.08 **	−0.05	−0.11 **	−0.08 **	-	−0.03
13. SES self- assessment	0.33 **	0.16 **	−0.19 **	0.20 **	0.19 **	0.28 **	0.18 **	0.2 **	0.15 **	0.04	−0.01	−0.03	-

Note. * *p* < 0.05; ** *p* < 0.01. Boys’ results above the diagonal, girls’ results below. CDI, Child Depression Inventory; SES, Socioeconomic Status.

**Table 3:** Multiple regression analysis for adolescent life satisfaction, stepwise method (N = 2239).

	Unstandardized Coefficients		Standardized Coefficients			Collinearity Statistics	
Significant Predictors by Steps	B	Standard Error	Beta (β)	t	*p*	Tolerance	VIF
(Constant)	5.08	1.25		4.06	*p* < 0.001		
1. Family support	0.49	0.03	0.44	16.85	*p* < 0.001	0.62	1.61
2. Depression (CDI)	−0.58	0.06	−0.24	−9.29	*p* < 0.001	0.65	1.53
3. SES self-assessment	0.78	0.16	0.11	5.02	*p* < 0.001	0.93	1.07
4. Future orientation	0.15	0.04	0.09	3.84	*p* < 0.001	0.80	1.25
5. Satisfaction with school	0.44	0.12	0.08	3.69	*p* < 0.001	0.81	1.23
6. Going to church	1.03	0.44	0.05	2.33	*p* < 0.05	0.99	1.01
7. Friend support	0.06	0.03	0.05	2.31	*p* < 0.05	0.79	1.27
R^2^ = 0.55							
*Excluded variables*							
Parental supervision					*p* > 0.05		
Shared dinner					*p* > 0.05		
Importance of religion					*p* > 0.05		
School achievement					*p* > 0.05		
Age					*p* > 0.05		
Sex					*p* > 0.05		

Note. CDI, Child Depression Inventory; SES, Socioeconomic Status; R2, R-squared, Coefficient of determination; B, Unstandardized regression coefficient; β, Standardized regression coefficient; t, Test statistics; p, Probability (significance); VIF, Variance Inflation Factor.

## Data Availability

The datasets generated during and/or analyzed during the current study are available from the author on reasonable request.
